# Tetramethylpyrazine inhibits ferroptosis in spinal cord injury by regulating iron metabolism through the NRF2/ARE pathway

**DOI:** 10.3389/fphar.2024.1503064

**Published:** 2024-11-15

**Authors:** Jingwei Tao, Jingya Zhou, Hanjie Zhu, Lin Xu, Jizhou Yang, Xiaohong Mu, Xiao Fan

**Affiliations:** ^1^ Orthopedic Surgery Center, Dongzhimen Hospital Beijing University of Chinese Medicine, Beijing, China; ^2^ Beijing University of Chinese Medicine, Beijing, China; ^3^ Department of Endocrinology, Capital Medical University Affiliated Beijing Hospital of Traditional Chinese Medicine, Capital Medical University, Beijing, China; ^4^ Shaoxing TCM Hospital Affiliated to Zhejiang Chinese Medical University, Shaoxing, China; ^5^ Bone and Joint Surgery, Qingdao Municipal Hospital, Qingdao, China

**Keywords:** tetramethylpyrazine, spinal cord injury, ferroptosis, iron metabolism, neuroprotection

## Abstract

**Background:**

Tetramethylpyrazine (TMP) is a natural alkaloid compound with antioxidant and neuroprotective effects. We hypothesized that TMP could exert neuroprotective effects by inhibiting ferroptosis through modulating iron metabolism, but its mechanism is unclear. Through *in vivo* and *in vitro* experiments, we have explored how TMP can regulate neurons’ iron metabolism through the NRF2/ARE pathway to Inhibit ferroptosis.

**Methods:**

In the *in vivo* experiment, the effects of TMP on nerve function and secondary spinal cord injury were observed through behavioral tests and morphology staining. Transmission electron microscopy, molecular biology tests and immunofluorescence staining were used to investigate the role of TMP in the regulation of iron metabolism and ferroptosis through the Nrf2/ARE pathway. Using *in vitro* experiments to investigate the mechanism of TMP in inhibiting ferroptosis through the Nrf2/ARE pathway.

**Results:**

Firstly, through *in vivo* experiments, we found that TMP improves motor function of rats with spinal cord injury, reduces spinal cord tissue damage and nerve cell death caused by secondary injury. Moreover, neuronal death and the formation of spinal cord cavities are inhibited by TMP. By regulating lipid peroxidation, TMP can inhibit mitochondrial damage and reduce ROS accumulation. Our study also demonstrated that TMP regulates iron metabolism through the NRF2/ARE pathway to inhibit ferroptosis and repair spinal cord injury. To further explore the regulatory mechanisms of TMP we down-regulating Nrf2 expression in subsequent *in vitro* experiments. We find that a key ferroptosis pathway, lipid peroxidation, can be regulated by TMP. Additionally, TMP inhibits iron overload-mediated ferroptosis by increasing Nrf2 transcriptional activity.

**Conclusion:**

A regulatory effect of TMP on the NRF2/ARE pathway was found in both *in vitro* and *in vivo* experiments. It promotes the transcription and translation of iron metabolizing and antioxidant molecules. Our study explored the inhibitory effect of TMP on ferroptosis from the iron metabolism pathway and provided new ideas for the treatment of SCI.

## 1 Introduction

It is known that mature mammalian spinal cord neurons are incapable of regenerating (Varadarajan et al., 2022). Therefore, spinal cord injury (SCI) has irreversible and significant damage to various essential physiological functions (Anjum et al., 2020). According to estimates provided by the World Health Organization, the annual incidence of SCI is approximately 500,000 individuals (Courtine and Sofroniew, 2019). The majority of the afflicted individuals experience paralysis that persists for several years (Alizadeh et al., 2019). Despite this, current medical interventions are not considered adequate for SCI (Tao et al., 2023). Microenvironmental imbalance is one of the obstacles encountered in spinal cord injury treatment. Researchers found that free radicals and heme accumulation can disrupt the microenvironment, inhibiting nerve repair and causing further damage to the spinal cord (Fan et al., 2018). Therefore, there is a pressing need for pharmaceuticals that could modulate the microenvironment to exert neuroprotective effectiveness.

A key pathological change in the microenvironment is ferroptosis caused by heme and free radical accumulation. Ferroptosis is iron-dependent nonapoptotic cell death distinct from autophagy, apoptosis, or necrosis. In contrast to other forms of programmed cell death, ferroptosis has unique morphological and biochemical characteristics (Dixon et al., 2012). It is manifested as mitochondria shrinkage and the emergence of membrane vesicles (Chen et al., 2021). In addition, the bioinformatics investigation revealed that ferroptosis-related genes were expressed differently in SCI patients (Qu et al., 2023). Disrupting the blood-spinal cord barrier leads to the accumulation of ferrous ions in neurons (Shah et al., 2018). Resulting an imbalance in the labile iron pool causes lipid peroxidation of PUFA in neuron membranes, which increases cell vulnerability to ferroptosis (Chen et al., 2020a). Thus, we surmise that SCI may be more susceptible to ferroptosis.

Developing drugs that regulate ferroptosis is an innovative direction for repairing spinal cord injuries. Tetramethylpyrazine (TMP) is an alkaloid naturally occurring in Ligusticum chuanxiong Hort’s dried rhizomes, a plant of the umbrella family (Lin et al., 2022). Several studies have shown that TMP has pharmacological actions that include antioxidants, suppressing Inflammation, regulating apoptosis, and modulating lipid metabolism (Hu et al., 2022; Li et al., 2021; Yang et al., 2021). Different pharmacological effects in turn interact with each other. It is extensively utilized in treating central nervous system diseases such as Ischemic cerebrovascular injury and neurodegenerative diseases (Feng et al., 2023; Meng et al., 2021; Zhou et al., 2019; Zhou et al., 2023). Additionally, TMP’s neuroprotective properties have been associated with its use in spinal cord injury treatment (Liu et al., 2022b). In our previous animal studies, we preliminarily explored the pharmacological role of TMP in regulating ferroptosis (Liu et al., 2024). However, previous experiments were less extensive and lacked cross-validation through *in vivo* and *in vitro* experiments. In regards to three major pathways regulating ferroptosis (1. PUFA and lipid metabolism pathway, 2. ROS-induced peroxidation damage, and 3. Iron metabolism pathway.), TMP’s pharmacological effects have not been researched. Due to a lack of in-depth research the mechanism by which TMP regulates ferroptosis is still unclear. According to recent studies, it has been proposed that the regulation of iron storage and iron export may serve as a new target to impede ferroptosis, hence offering potential neuroprotective benefits (Stockwell et al., 2017). Nevertheless, none of studies have been undertaken on this topic in spinal cord injury treatment. Iron metabolism pathway of ferroptosis needs more in-depth study. In our study, we targeted the iron metabolism pathway, one of the three major regulatory pathways of ferroptosis. Through *in vivo* and *in vitro* experiment, we verified that TMP regulates iron metabolism to inhibit ferroptosis through the Nrf2/ARE pathway.

The transcription factor NRF2, a nuclear factor encoded erythroid two like 2 (NFE2L2), is a member of the basic leucine zipper Cap’n'Collar (CNC) subfamily (He et al., 2020). NRF2 can attach to the promoter region’s antioxidant response element (ARE) to regulate gene expression (Liu et al., 2022a). Proteins encoded by these genes exert cytoprotective effects by acting as antioxidants and anti-inflammatory agents (Ucar et al., 2021; Zhuang et al., 2019). Recent studies have shown that NRF2 and its target genes are also related to iron storage, export, and metabolism (Song and Long, 2020). Regulating iron metabolism through the NRF2/ARE pathway may be essential in inhibiting ferroptosis of SCI. TMP has antioxidant and lipid metabolism regulatory effects. NRF2 plays a key transcriptional regulatory role in lipid peroxidation and iron metabolism. Thus, we suggest that TMP can regulate ferroptosis through the NEF2/ARE pathway.

In this study, we investigated the inhibitory effects of TMP on ferroptosis. Through *in vivo* and *in vitro* experiments, we have explored how TMP can regulate neurons’ iron metabolism through the NRF2/ARE pathway to provide new insight for treating SCI.

## 2 Materials and methods

### 2.1 Animals

Forty-five female Sprague-Dawley (SD) rats weighing 200–240 g were purchased from SiPeiFu (Beijing) Biotechnology Co. Ltd. and were housed in the Yifu research center of the Beijing University of Chinese Medicine. Rats were kept in a temperature range of 20°C–22°C and a humidity range of 40%–50%. The animals were also provided with free food, water, and a 12-hour light/dark cycle. The Beijing University of Chinese Medicine Ethics Committee approved this study under the approval number BUCM-2022042104–2098.

### 2.2 SCI model and drug treatment

Forty-five rats were randomly assigned into three groups: sham, model, and TMP (40 mg/kg, 80 mg/kg, 160 mg/kg). The experiment was started after 1 week of acclimatization feeding. Rats were given 1% pentobarbital (40 mg/kg) to make them anesthetized, the T9 vertebral plate and spinous processes were removed, and spinal cord contusion injury was inflicted by free-falling a 10 g weight percussion rod at 2.5 cm dorsal to the spinal cord using a custom-made spinal cord striker (Hualianke Biotechnology Co.). Penicillin 200,000 U/day was administered subcutaneously for 3 days postoperatively in each group to prevent infection, and bladder massage was performed twice daily in the morning and evening to assist urination until voluntary urination was resumed.

Sham group was sutured immediately after exposing the spinal cord. Each day, the same amount of saline was administered intraperitoneally for 7 days after spinal cord injury. Upon successful modeling, daily doses of saline were administered intraperitoneally in the model group for 7 days after spinal cord injury. And then, we set up 3 dosages to screen the appropriate administration dose of TMP (40 mg/kg, 80 mg/kg, 160 mg/kg, intraperitoneal injection for 7 days after spinal cord injury). The injection dose was based on the conversion of the body’s surface area of humans and rats and reference to the effective dose of intraperitoneal injection of TMP in rats as reported in the literature (Hu et al., 2016). In subsequent experiments, rats in the TMP group were given TMP intraperitoneally (80 mg/kg, intraperitoneal injection for 7 days after spinal cord injury) daily before surgery and after successful modeling. Intraperitoneal administration is very common in rodent testing, and the advantages of intraperitoneal include ease of administration, rapidity, low animal stress, and large volume of administration. Faster absorption and higher bioavailability of intraperitoneal administration compared to oral administration. Therefore, we chose to administer the drug by Intraperitoneal administration.

### 2.3 Behavioral tests

Basso, Beattie, and Bresnahan scale scores (BBB), and inclined plane tests were used to investigate the recovery of motor function. At the 0, 1, 3, 7, 14, 21, 28, 35and 42 days after treatment, two experimenters independently used the BBB scoring scale (21 represents normal hindlimb locomotor function, while 0 represents no hindlimb locomotor function), and the inclined plane tests (observing the maximum angle of the rats that stayed on an incline for 5 s) to evaluate the locomotor function. The average of both individuals’ scores will be used as the final statistical value.

### 2.4 Histological stain

On the 42nd day and 7th day after spinal cord injury, rat spinal cord tissues of 2 cm in length were fixed in 4% paraformaldehyde (PFA). We prepared paraffin sections with a thickness of 5 μm from the samples embedded in paraffin. The 42nd day after spinal cord injury, tissues paraffin sections were stained with Hematoxylin-Eosin (HE) stain (Solarbio) and Nissl stain (Solarbio) according to the kit instructions. The 7th day after spinal cord injury, spinal cord tissues paraffin sections were stained with Prussian Blue Iron stain (Solarbio) according to the kit instructions. The area of spinal cord defects in HE-stained images and the average optical density of positive staining for Nissl body in spinal cord anterior horn neuron was analyzed using ImageJ.

### 2.5 Iron content assay

On the 7th day after spinal cord injury, we homogenized 0.1 g of rat spinal cord tissue in an ice bath. The supernatant was taken and assayed for iron content according to the Iron Assay Kit (Abcam) instructions, and the absorbance was read at 562 nm.

### 2.6 Transmission electron microscopy

On the 7th day after spinal cord injury, fresh spinal cord tissue was trimmed into 1 mm^3^ pieces and stored in a glutaraldehyde electron microscope fixative. 1% osmium acid was fixed at room temperature for 2 h to avoid lighting, and gradient dehydrated at room temperature. The embedded plates were polymerized. The ultrathin slicer prepared ultrathin slices of 60–80 nm, and the pieces were fished with 150 mesh copper mesh of film. The copper mesh was stained with a 2% uranyl acetate-saturated alcohol solution. Image analysis was performed using a transmission electron microscope.

### 2.7 Reactive oxygen species (ROS) fluorescent stain

On the 7th day after spinal cord injury, sucrose solutions of 15% and 30% dehydrate spinal cord tissue gradually. Spinal cord tissue was embedded with an OCT embedding agent (Sakura), and frozen sections were prepared. The autofluorescence quencher (Servicebio) was added. Add the ROS staining solution (Sigma) and incubate at 37° for 30 min in the thermostat.

### 2.8 Cell culture

We cultured VSC4.1 spinal cord anterior horn motoneuronoma cells (Otwo Biotech) in DMEM supplemented with 1% penicillin/streptomycin and 10% fetal bovine serum. Cells were cultivated at 37°C in 5% CO2 and saturated humidity. Using ammonium iron (III) citrate (FAC) to construct an *in vitro* iron overload ferroptosis model (Feng et al., 2021). The cells were incubated for 24 h with TMP or FAC. We apply the CCK-8 assay to ascertain the optimal dosage for FAC (10, 20, 50, 100, 150, or 200 μM) and TMP (10, 20, 50, 100, 150, or 200 μM) in the VSC4.1 cells. NRF2 siRNA and NC siRNA were purchased from Sangon Biotech Ltd. Cells were incubated for 24 h with Lipofectamine 3,000 (Thermo Fisher Scientific) mix. Western blot (WB) was used to confirm the effectiveness of transfection. We incubated the cells with the appropriate dose of FAC (10 μM) for 24 h to construct an iron overload-induced ferroptosis model. Then, the cells were next incubated for 24 h with TMP (100 μM) in preparation for further experiments.

### 2.9 Cell viability assay

Cell viability was assayed in each group according to the experimental protocol described in the Cell Counting Kit-8 assay kit (CCK-8) (Solarbio Life Science).

### 2.10 Elisa

Collection of cells for preparation of extracts solution, and the GSH-Px ELISA Kit was used to detect changes in GSH-Px expression according to the instructions.

### 2.11 Superoxide dismutase (SOD) activity and malondialdehyde (MDA) content assay

Add 1 mL of extraction reagent to rat spinal cord tissue or cells, centrifuge at 12,000 rpm, and extract the supernatant. Follow the instructions provided by the MDA content assay kit (Solarbio) and SOD activity assay kit (Solarbio). Calculation of SOD and MDA content.

### 2.12 Western blot

Spinal cord tissues on the 7th day after spinal cord injury or cells were lysed on ice, centrifugated, and prepared with an extract solution. Determination of protein concentrations using bicinchoninic acid (BCA) protein quantification method assay kit (Solarbio). Add 8 μL of processed sample to each well and separate by electrophoresis. The polyvinylidene fluoride (PVDF) membrane transfer was carried out at 4°C. The PVDF membrane was blocked using TBST and incubated with a primary antibody. The type of primary antibodies were: FTL (1:1000, Abcam, Cat# ab69090), FTH1 (1:1000, Abcam, Cat# ab183781), FPN1 (1:1000, Abcam, Cat# ab239511), NRF2 (1:5000, Abcam Cat# ab92946), GPX4 (1:2000, Abcam Cat# ab92946), HO-1 (1:5000, Abcam Cat# ab13243). A secondary antibody labeled with horseradish peroxidase (HRP) was incubated on the membranes. Color rendering and exposure were performed using a fully automated chemiluminescence analyzer. Use of ImageJ software for Grayscale data analysis. FTH, FTL, and FPN1 was the marker of iron metabolism. NRF2 is a key transcription factor in our study. HO-1 and GPX4 is the lipid peroxidation marker of ferroptosis.

### 2.13 Quantitative real-time polymerase chain reaction

Rat spinal cord on the 7th day after spinal cord injury were used to extract total RNA in accordance with the experimental procedure included in the total RNA extraction kit (Solarbio). To obtain cDNA, reverse transcription was carried out utilizing a universal reverse transcription reaction kit (Yeasen). An RT-PCR fluorescence quantification kit (Yeasen) was used to determine gene expression. Normalization of data using the 2^−ΔΔCT^ method. We summarize the primer sequences in Table 1.

**TABLE 1:** The primer sequences.

Gene	Primer sequence	
*GAPDH*	Forward	GGC​AAG​TTC​AAC​GGC​ACA​G
	Reverse	CGC​CAG​TAG​ACT​CCA​CGA​CA
*FTL*	Forward	AAC​CAC​CTG​ACC​AAC​CTC​CGT​AG
	Reverse	CAA​AGA​GAT​ACT​CGC​CCA​GAG​ATG​C
*FTH1*	Forward	AAC​CAG​CGA​GGT​GGA​CGA​ATC​T
	Reverse	AGG​TAA​TGC​GTC​TCA​ATG​AAG​TCA​CA
*FPN*	Forward	CAC​CAC​AGG​ATA​TGC​TTA​CAC​TCA​GG
	Reverse	GAG​AAC​AGA​CCA​GTC​CGA​ACA​AGG
*GPX4*	Forward	GCA​GGA​GCC​AGG​AAG​TAA​TCA​AGA​A
	Reverse	TAG​CAC​GGC​AGG​TCC​TTC​TCT​ATC
*HO1*	Forward	CTC​GCA​TGA​ACA​CTC​TGG​AGA​TGA​C
	Reverse	TGT​TGA​GCA​GGA​AGG​CGG​TCT​TA
*NRF2*	Forward	TGC​CTT​CCT​CTG​CTG​CCA​TTA​GT
	Reverse	ACC​GTG​CCT​TCA​GTG​TGC​TTC​T

### 2.14 Immunofluorescence staining

Preparation of cell crawls or paraffin sections were processed for antigen repair. Incubate samples with primary antibodies: NeuN (1: 200, Proteintech Cat# 66836-1-Ig), NRF2 (1: 200, Proteintech Cat# 16396-1-AP), GPX4 (1: 200, Abcam Cat# ab92946), HO-1 (1: 200, Abcam Cat# ab13243) at 4°C overnight. A fluorescent secondary antibody working solution was added to the samples and incubated for 2 hours at 37°C. A fluorescence quenching agent was used to block the samples. Photographs were taken using an inverted microscope imaging system (Nikon) and a confocal laser scanning microscope (Leica). Observation of protein expression in the anterior horn of the spinal cord. Image analysis using ImageJ. The 42nd day after spinal cord injury, tissues paraffin sections underwent NeuN immunofluorescence staining. ImageJ was used to analysis the relative fluorescence intensity of NeuN to assessment of nerve cell survival. The 7nd day after spinal cord injury, tissues paraffin sections underwent NRF2, HO-1 immunofluorescence staining. ImageJ was used to analysis the relative fluorescence intensity of NRF2 and HO-1.

### 2.15 Flow cytometry

Cells were collected and resuspended by adding 1 × binding buffer and adjusting its concentration to 1–5 × 10^6^/mL. In a 5 mL tube, mix 100ul of cell suspension with 5 μL of Annexin V/Alexa Fluor 647. Incubate away from light for 5 min. Add 10 μL of 20ug/mL propidium iodide solution (PI) and 400ul PBS. A flow cytometer was used to perform the assays. FlowJo 10.9.0 was used to analyze the data.

### 2.16 Luciferase assays

Firefly and sea kidney luciferase substrate dilution using buffers according to the experimental procedure provided in the ARE Dual-Luciferase Reporter Gene Assay Kit (Yeasen). Cells of each group were transfected using ARE Luciferase Reporter Plasmid (Yeasen, Shanghai). Wild-type ARE sequences: GGCTTAATCAC, mutant-type ARE sequences: GGCTGATTCAT. Dilute 0.15ug of reporter gene vector with 10ul of serum-free medium, add Hieff TransTM dilution reagent and incubate for 5 min. Diluted Hieff TransTM reagent is added dropwise to the reporter gene vector solution to form the DNA-Hieff TransTM mixed solution. The mixture was added to 96-well plates, incubated for 6h, and replaced with a complete medium. Two fluorescein substrates were added separately to the cells to detect the activity of firefly and sea kidney luciferase. The relative luciferase activity is the firefly luciferase assay values ratio to sea kidney luciferase assay values. The relative luciferase activity is the firefly luciferase assay values ratio to sea kidney luciferase assay values.

### 2.17 Statistical analysis

Software programs SPSS 20.0 and GraphPad Prism 10.0 were used to analyze all of the data. The data were transformed into mean values ± SD for statistical analysis. We used one-way ANOVA for comparisons when the experimental results followed a normal distribution. Post hoc analyses were performed utilizing either the Games-Howell or LSD test. A Kruskal–Wallis compares the results of experiments that do not follow a normal distribution. P < 0.05 is a significant result.

## 3 Results

### 3.1 TMP inhibits spinal cord secondary injury and improves motor function in rats, 80 mg/kg is the appropriate dose for TMP

**FIGURE 1:**
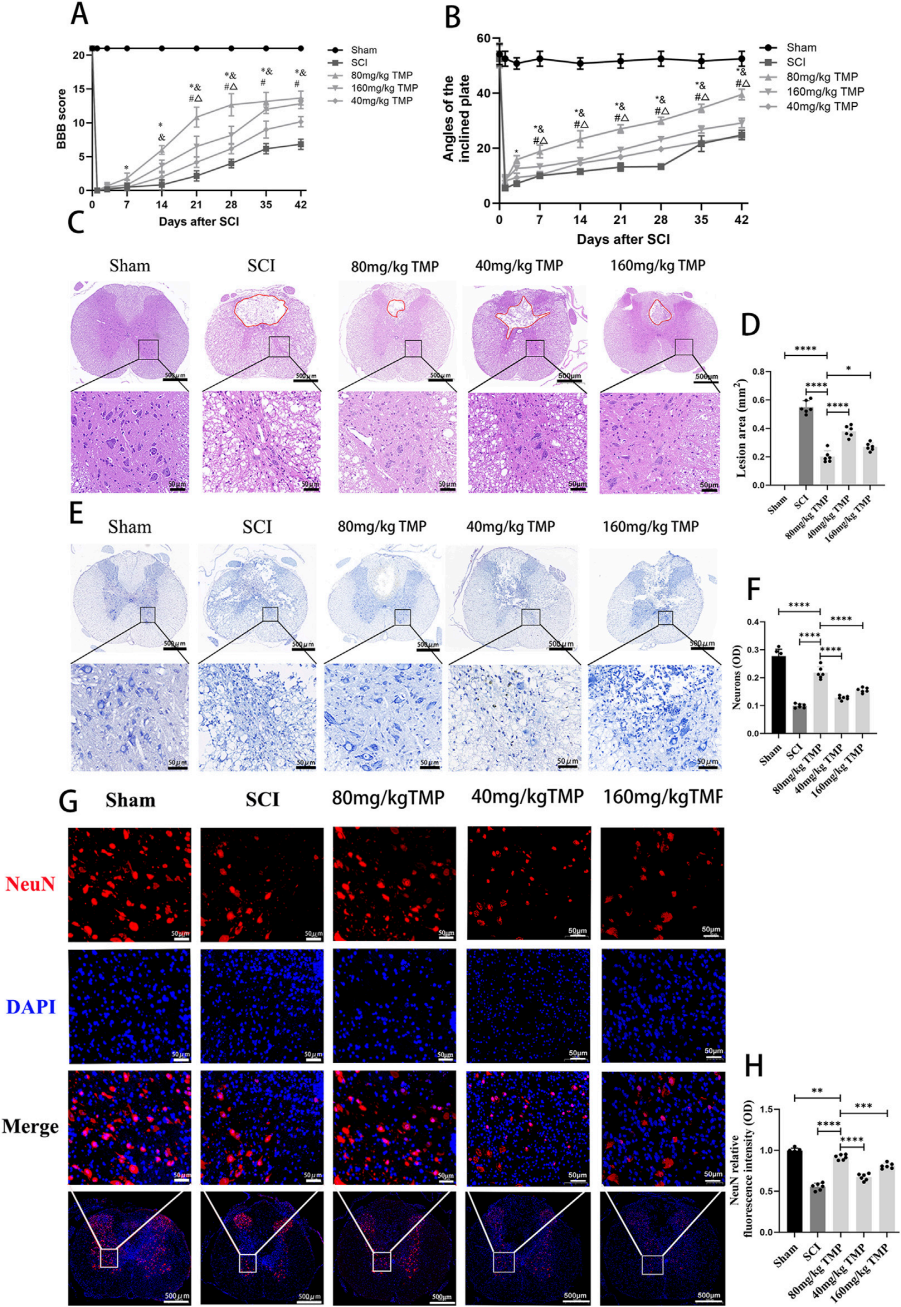
TMP inhibits SCI and improves motor function in rats. **(A)** Results of BBB scores (n = 6, ‾x ± s. Statistical analysis was performed with a Kruskal–Wallis test and Dunn’s test. Compare with Sham group, *P < 0.05. Compare with SCI group, & *P* < 0.05. Compare with 40 mg/kg TMP group, #*P* < 0.05. Compare with 160 mg/kg TMP group, △*P* < 0.05). **(B)** Results of inclined plane tests (n = 6, the data represent means ± SD. Statistical analysis was performed with a Kruskal–Wallis test and Dunn’s test. Compare with Sham group, *P < 0.05. Compare with SCI group, & *P* < 0.05. Compare with 40 mg/kg TMP group, #*P*< 0.05. Compare with 160 mg/kg TMP group, △*P* < 0.05). **(C)** HE staining (n = 6, Scale bar in above image: 500 μm, Scale bar in below image: 50 μm) and **(D)** analysis of lesion area (Statistical analysis was performed with a one-way ANOVA and LSD test, F (4, 25) = 203.6, P < 0.0001. *: P < 0.05; ****: P < 0.0001). The red area represents the lesion. **(E)** Nissl staining (n = 6, Scale bar in above image: 500 μm, Scale bar in below image: 50 μm) and **(F)** average optical density of Nissl body in anterior horn neurons of the spinal cord (Statistical analysis was performed with a one-way ANOVA and LSD test, F (4, 25) = 118.3, P < 0.0001. ****: P < 0.0001). **(G)** NeuN immunofluorescence staining (n = 6, Scale bar in below image: 500 μm, Scale bar in above three image: 50 μm) and **(H)** NeuN relative fluorescence intensity in anterior horn neurons of the spinal cord (Statistical analysis was performed with a one-way ANOVA and LSD test, F (4, 25) = 146.7, P < 0.0001. ****: P < 0.0001). The data represent means ± SD.

To examine the impact of TMP on impeding secondary spinal cord injury and enhancing motor function in rats after SCI, we tracked the hindlimb motor function in rats 1, 3, 7, 14, 21, 28, 35, and 42 days later. Pathologic staining was used to observe the lesion areas of spinal cord tissue defect and morphology of neurons in the anterior horn of the spinal cord on the 42nd day in the rats. The rats in the sham group had normal motor function. The rats in the SCI and TMP groups had almost no motor function in the hind limbs on the 1st day after SCI. After that, there was a trend for motor function to recover gradually. The BBB scores and inclined plane test results suggested no difference in motor function in SCI and TMP groups on days 1 and 3 after spinal cord injury. According to the BBB scores and inclined plane tests on days 7, 14, 21, 28, 35, and 42, TMP treatment significantly improves motor function in rats. Among them, 80 mg/kg TMP improved motor function better than 40 mg/kg and 160 mg/kg (Figures 1A, B). HE staining indicated that on day 42 after SCI. it was observed that the spinal cord of rats in the sham group was structurally intact. The structure of motor neurons in the anterior horn of the spinal cord and nerve cell morphology was intact. In the SCI group, there were obvious cavities in the spinal cord tissue. The anterior horn motor neuron region of the spinal cord was missing, with local cavities and fibrous connective tissue infiltration. The area of spinal cord defect was significantly smaller in the TMP group. Compare with the 40 mg/kg TMP group and the 80 mg/kg TMP group, the 160 mg/kg TMP group showed a higher survival of motor neurons in the anterior horn of the spinal cord and a smaller cavity areas (Figures 1C, D). Nissl staining and immunofluorescence staining on day 42 after injury suggested that the survival of neurons in anterior horn neurons of the spinal cord was significantly higher in the TMP group, and the survival of neurons in 80 mg/kg TMP group was better than 40 mg/kg TMP group and 160 mg/kg TMP group (Figures 1E–H). Therefore, TMP can prevent secondary SCI in rats, improve motor performance, and have neuroprotective effects. 80 mg/kg is the appropriate TMP dose. And we chose 80 mg/kg as the appropriate dose of TMP for subsequent experiments.

### 3.2 TMP exerts neuroprotective effects by inhibiting spinal cord neuron ferroptosis in rats

**FIGURE 2:**
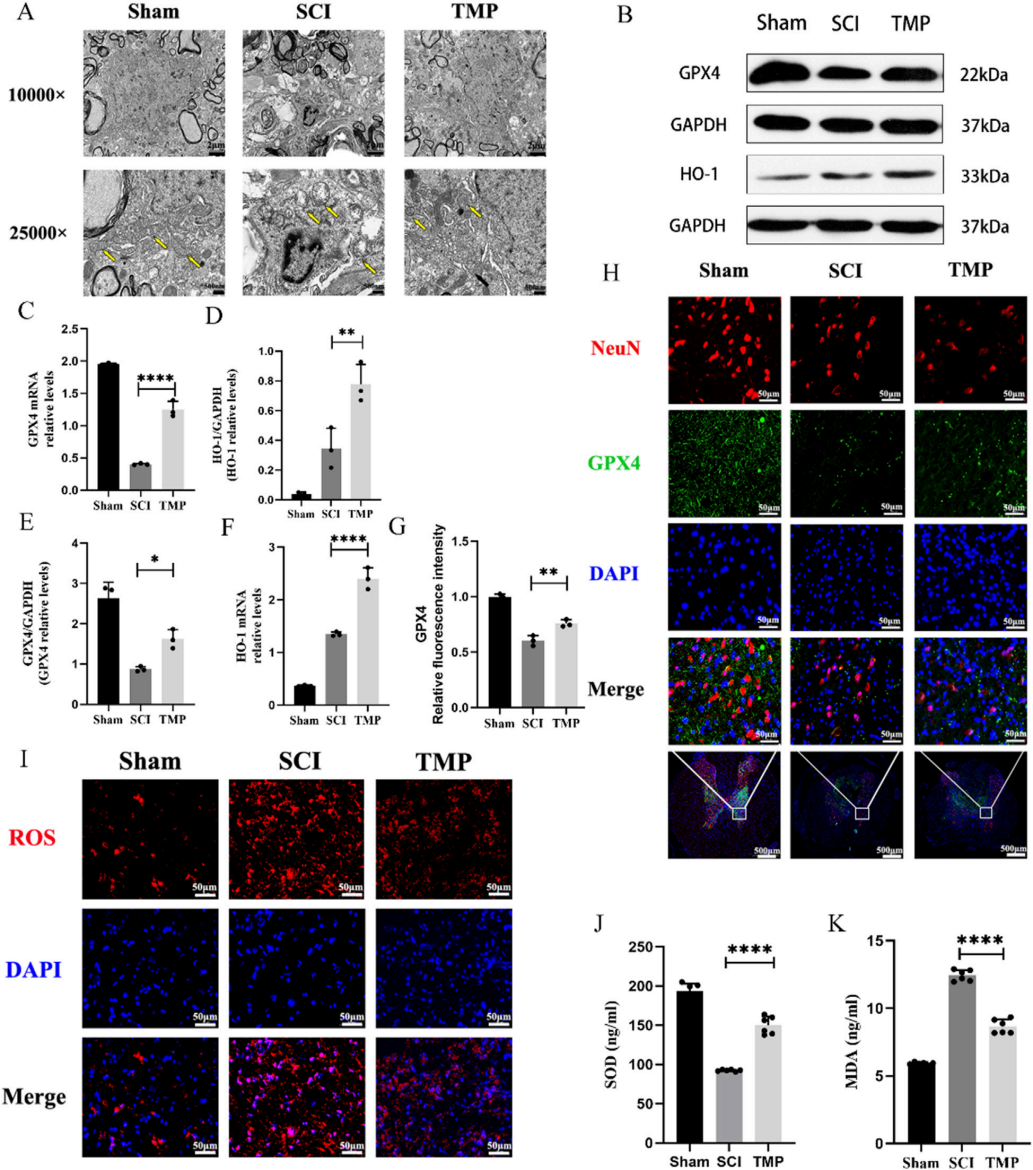
TMP exerts neuroprotective effects by inhibiting spinal cord neuron ferroptosis in rats. **(A)** Transmission electron microscopy results for each group, the yellow arrows show mitochondria (n = 3, Scale bar in above image: 2 μm, Scale bar in below image: 500 nm). **(B)** Western blot and **(C, D)** quantitative analysis of GPX4, HO-1 (n = 3, Statistical analysis was performed with a one-way ANOVA and LSD test, GPX4: F (2, 6) = 31.53, P = 0.0007, HO-1: F (2, 6) = 33.69, *p* = 0.0005. **: P < 0.01; ****: P < 0.0001). **(E, F)** qRT-PCR results for GPX4, HO-1 (n = 3, Statistical analysis was performed with a one-way ANOVA and LSD test, GPX4: F (2, 6) = 334.9, P< 0.0001, HO-1: F (2, 6) = 212.9, P < 0.0001. *: P < 0.05; **: P < 0.01; ****: P < 0.0001). **(H)** Immunofluorescence staining for GPX4 and NenN in anterior horn neurons of the spinal cord (n = 3, Scale bar in below image: 500 μm, Scale bar in above three image: 50 μm). **(G)** Relative fluorescence intensity for GPX4 (n = 3, statistical analysis was performed with a one-way ANOVA and LSD test, F (2, 6) = 88.32, P < 0.0001. **: P < 0.01). **(I)** ROS fluorescent stain result for each group (n = 3, Scale bar: 50 μm). **(J)** SOD and **(K)** MDA levels in each group (n = 3, Statistical analysis was performed with a one-way ANOVA and LSD test, SOD: F (2, 15) = 216.1, P < 0.0001, MDA: F (2, 15) = 424.6, P < 0.0001. ****: P < 0.0001). The data represent means ± SD.

Disruption of mitochondrial membrane structure is an important morphological feature of ferroptosis (Ma et al., 2022). Seven days after SCI, transmission electron microscopy results indicated that the sham group’s mitochondrial bilayer membrane structure was intact and morphologically intact. However, those in the SCI group showed disorganized mitochondrial structure and decreased mitochondrial ridges. Compared to the SCI group, the TMP group’s mitochondrial substructure was more completed and showed the structure of the mitochondrial bilayer membrane (Figure 2A). Ferroptosis is characterized by dysregulation of lipid peroxides, such as ROS, SOD, MDA, HO-1, and GPX4. ROS and MDA cause lipid peroxidation damage in nerve cells. GPX4, SOD and HO-1 have an antioxidant effect. The results of WB and qRT-PCR indicated that TMP treatment for 7 consecutive days significantly increased GPX4 expression, the markers of ferroptosis. The TMP treatment also increased HO-1 levels (Figures 2B–F). Immunofluorescence results revealed that there is a significant increase in the expression of GPX4 in neurons of the spinal cord anterior horn following TMP administration (Figures 2G, H), consistent with the outcomes of WB and qRT-PCR. ROS fluorescence staining indicated that the TMP group had lower ROS expression than the SCI group but higher ROS expression than the Sham group in spinal cord (Figure 2I). We similarly found that TMP was able to lower the levels of MDA while raising the expression of SOD, which is consistent with changes in ROS expression (Figures 2J, K). According to the studies above, TMP prevents neuronal ferroptosis from the lipid peroxidation pathway. Based on these findings, we conducted follow-up experiments to investigate the regulatory mechanism of TMP on ferroptosis.

### 3.3 TMP inhibits ferroptosis by modulating iron metabolism pathways through the NRF2/ARE pathway

**FIGURE 3:**
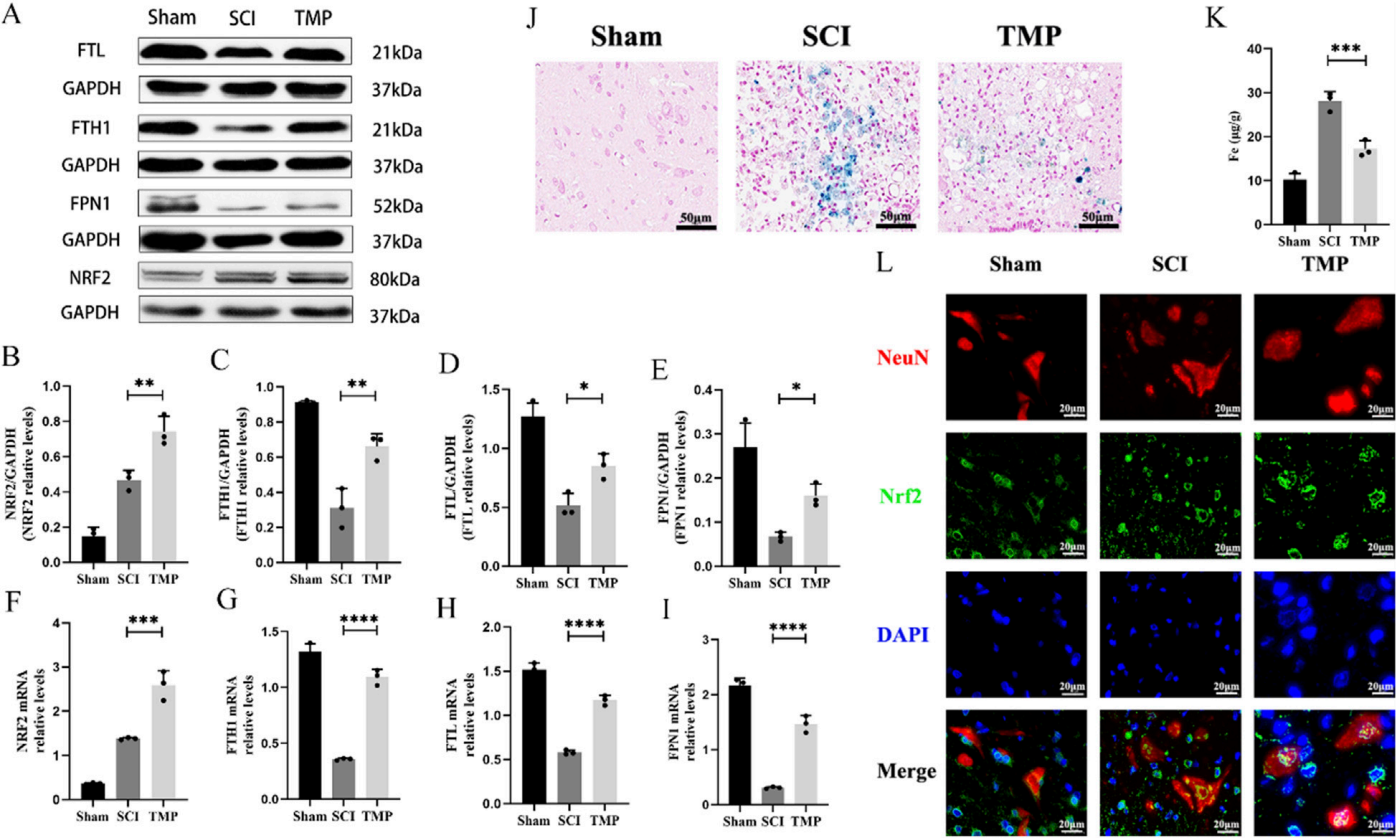
TMP inhibits ferroptosis by modulating iron metabolism pathways through the NRF2/ARE pathway in rats. **(A)** Western blot and **(B–E)** relative NRF2, FTH1, FTL, and FPN1 levels in rats (n = 3, Statistical analysis was performed with a one-way ANOVA and LSD test, NRF2: F (2, 6) = 61.68, P< 0.0001, FTH1: F (2, 6) = 46.60, *p* = 0.0002, FTL: F (2, 6) = 36.50, P = 0.0004, FPN1: F (2, 6) = 25.20, P = 0.0012. *: P < 0.05; **: P < 0.01; ****: P < 0.0001). **(F–I)** qRT-PCR assay for NRF2, FTH1, FTL, and FPN1 in rats (n = 3, Statistical analysis was performed with a one-way ANOVA and LSD test, NRF2: F (2, 6) = 104.0, P < 0.0001, FTH1: F (2, 6) = 240.8, P < 0.0001, FTL: F (2, 6) = 206.5, P < 0.0001, FPN1: F (2, 6) = 196.6, P < 0.0001. *: P < 0.05; **: P < 0.01; ****: P < 0.0001). **(J)** Prussian Blue stain (n = 3). **(K)** Iron content assay (n = 3, Statistical analysis was performed with a one-way ANOVA and LSD test, F (2, 6) = 72.08, P < 0.0001. ***: P < 0.001). **(L)** Immunofluorescence staining for NRF2 and NenN in anterior horn neurons of the spinal cord (n = 3, Scale bar: 20 μm). The data represent means ± SD.

It has been shown that NRF2 regulates the transcription of most ferroptosis genes through binding to antioxidant response elements (Yan et al., 2023). These include the key iron metabolism gene FTH1, FTL and FPN1. We observed TMP’s regulation of iron metabolism levels through the NRF2/ARE pathway. The WB and qRT-PCR analysis results indicated that TMP can increase NRF2 expression. Iron metabolism-related molecules FTH1, FTL, and FPN1 were found at higher levels in the TMP group compared to the SCI group and at lower levels in the Sham group (Figures 3A–I). According to the Prussian blue staining and iron content assay, the iron concentration of the spinal cord injury region in the TMP group was lower than that of the SCI group and higher than that of the Sham group (Figures 3J, K). As shown by immunofluorescence staining, Nrf2 was more abundant in the nuclei of neurons in the TMP group (Figure 3L). In contrast, it was more abundant in the cytoplasm of neurons in the Sham group. Based on these findings, we have preliminarily demonstrated that TMP regulates iron metabolism and NRF2/ARE pathway.

### 3.4 TMP improves the viability of ferroptosis VSC4.1 cells via the NRF2 pathway

**FIGURE 4:**
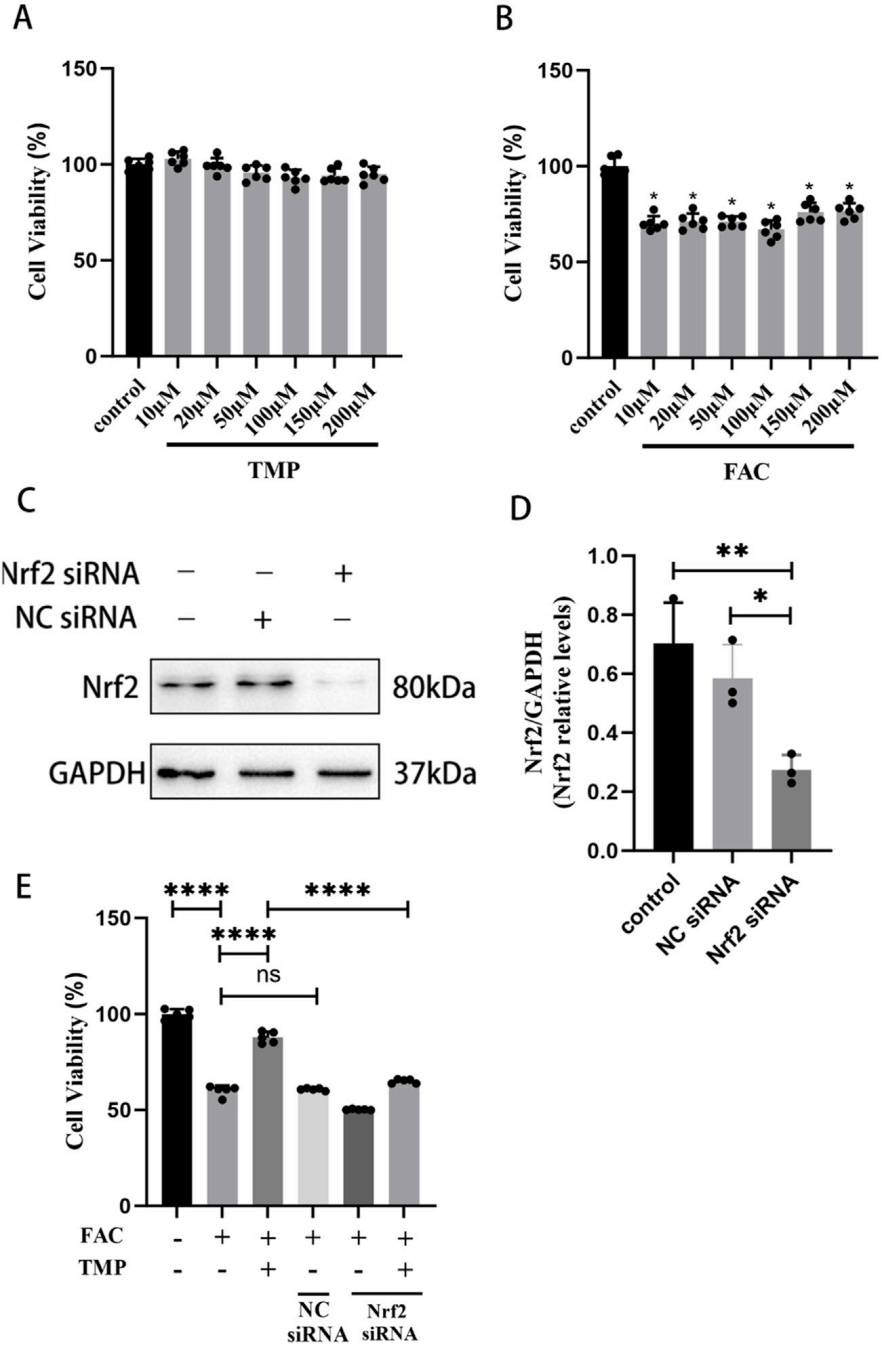
TMP improves the viability of ferroptosis VSC4.1 cells via the NRF2 pathway. **(A)** CCK-8 assay screens TMP addition concentration (Concentration gradient: 10, 20, 50, 100, 150, or 200μM, n = 6. Statistical analysis was performed with a one-way ANOVA, F (6, 35) = 5.650 P > 0.05). **(B)** CCK-8 assay screens FAC addition concentration (Concentration gradient: 10, 20, 50, 100, 150, or 200 μM, n = 6. Statistical analysis was performed with a one-way ANOVA and LSD test, F (6, 35) = 41.51, P < 0.0001. *: P < 0.05). **(C, D)** Western blot detection of NRF2siRNA transfection efficiency (n = 3, Statistical analysis was performed with a one-way ANOVA and LSD test, F (2, 6) = 12.66, P = 0.0070. *: P < 0.05; **: P < 0.01). **(E)** CCK-8 assay for each group of cell viability (n = 6, Statistical analysis was performed with a one-way ANOVA and LSD test, F (5, 24) = 447.4, P < 0.0001. ****: P < 0.0001, ns: the difference was not statistically significant). The data represent means ± SD.

For the first step of the *in vitro* experiment, we utilized the CCK-8 assay to measure appropriate concentrations of FAC and TMP. According to the CCK-8 assay, the effect of TMP (10, 20, 50, 100, 150, or 200 μM) on cell viability was not significantly different from that of the control group (Figure 4A). Compared to the control group, the FAC group’s cellular viability (10, 20, 50, 100, 150, or 200 μM) was lower (Figure 4B). No significant difference in cellular viability between the different doses. Therefore, we chose the minimum dose 10 μM as the dosing of FAC to construct an iron overload ferroptosis cell model and 100 μM as the dosing of TMP to inhibit cell ferroptosis. WB was used to screen successful transfected VSC4.1 cells for further experiments (Figures 4C, D). CCK-8 assay suggested that TMP could inhibit ferroptosis and improve cellular viability through the NRF2 pathway. The cell viability of the ferroptosis cell model was significantly lower than that of the control group. TMP was able to increase the cell viability of the ferroptosis cell model. Silencing of NRF2 reversed the effect of TMP on ferroptosis cell. (Figure 4E). This suggests that TMP is able to inhibit ferroptosis through the NRF2 pathway.

### 3.5 TMP exerts neuroprotective effects by inhibiting ferroptosis in VSC4.1 cells through the NRF2 pathway

**FIGURE 5:**
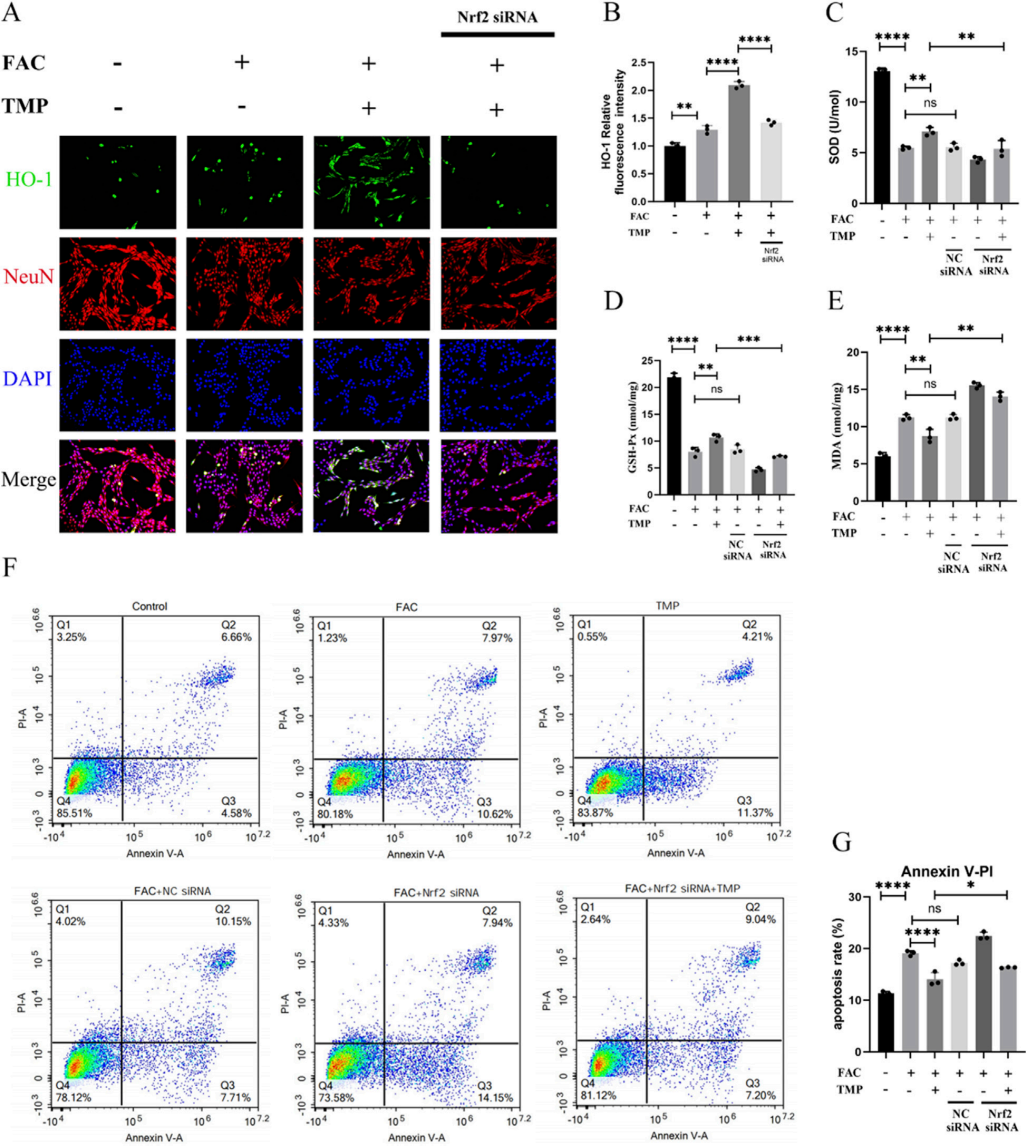
TMP exerts neuroprotective effects by inhibiting ferroptosis in VSC4.1 cells through the NRF2 pathway. **(A)** Immunofluorescence staining for HO-1 and NenN for each group (n = 3, Scale: 50 μm). **(B)** Relative fluorescence intensity for HO-1 in VSC 4.1 Cell (n = 3, statistical analysis was performed with a one-way ANOVA and LSD test, F (3, 8) = 172.6, P < 0.0001. **: P < 0.01, and ****: P < 0.0001). **(C)** Expression of SOD and **(E)** MDA in each group (n = 3, statistical analysis was performed with a one-way ANOVA and LSD test, SOD: F (5, 12) = 152.7, P < 0.0001, MDA: F (5, 12) = 115.3, P < 0.0001. **: P < 0.01, and ****: P < 0.0001, ns: there was no statistical difference). **(D)** Elisa detection of GSH-Px Expression (n = 3, statistical analysis was performed with a one-way ANOVA and LSD test, F (5, 12) = 258.5, P< 0.0001. **: P < 0.01, ***: P < 0.001, and ****: P < 0.0001, ns: there was no statistical difference). **(F, G)** Flow cytometry analysis of AnnexinV/PI assay (n = 3, statistical analysis was performed with a one-way ANOVA and LSD test, F (5, 12) = 138.2, P < 0.0001. *: P < 0.05, ****: P < 0.0001, ns: there was no statistical difference). The data represent means ± SD.

Dysmetabolism of lipid peroxides such as HO-1, SOD, MDA and GSH-Px is an important feature of ferroptosis. TMP exerts a neuroprotective effect by regulating the expression of ferroptosis lipid peroxidation markers through the NRF2 pathway. To examine the function of TMP, we used NRF2 siRNA to downregulate the transcription factor NRF2. According to immunofluorescence staining, HO-1 expression was shown to be lower in the ferroptosis group compared to the TMP group. Downregulation of NRF2 reversed the upregulation of HO-1 by TMP. (Figures 5A, B). Similarly, the expression of SOD and GSH-Px was lower in the ferroptosis group, and the expression of MDA was higher in the ferroptosis group. TMP upregulated the expression of SOD and GSH while downregulated the expression of MDA. Silencing of NRF2 reverses the ability of TMP to regulated the levels of SOD, GSH-Px, and MDA (Figures 5C–E). According to the AnnexinV/PI assay, the ferroptosis group had an increased apoptosis rate than control group. Silencing of NRF2 reverses the inhibitory effect of TMP on apoptosis in ferroptosis cell model (Figures 5F, G). Therefore, TMP can inhibit ferroptosis by regulating lipid peroxidation through the NRF2 pathway.

### 3.6 TMP inhibits ferroptosis in VSC4.1 cells by regulating iron metabolism through the NRF2/ARE pathway

**FIGURE 6:**
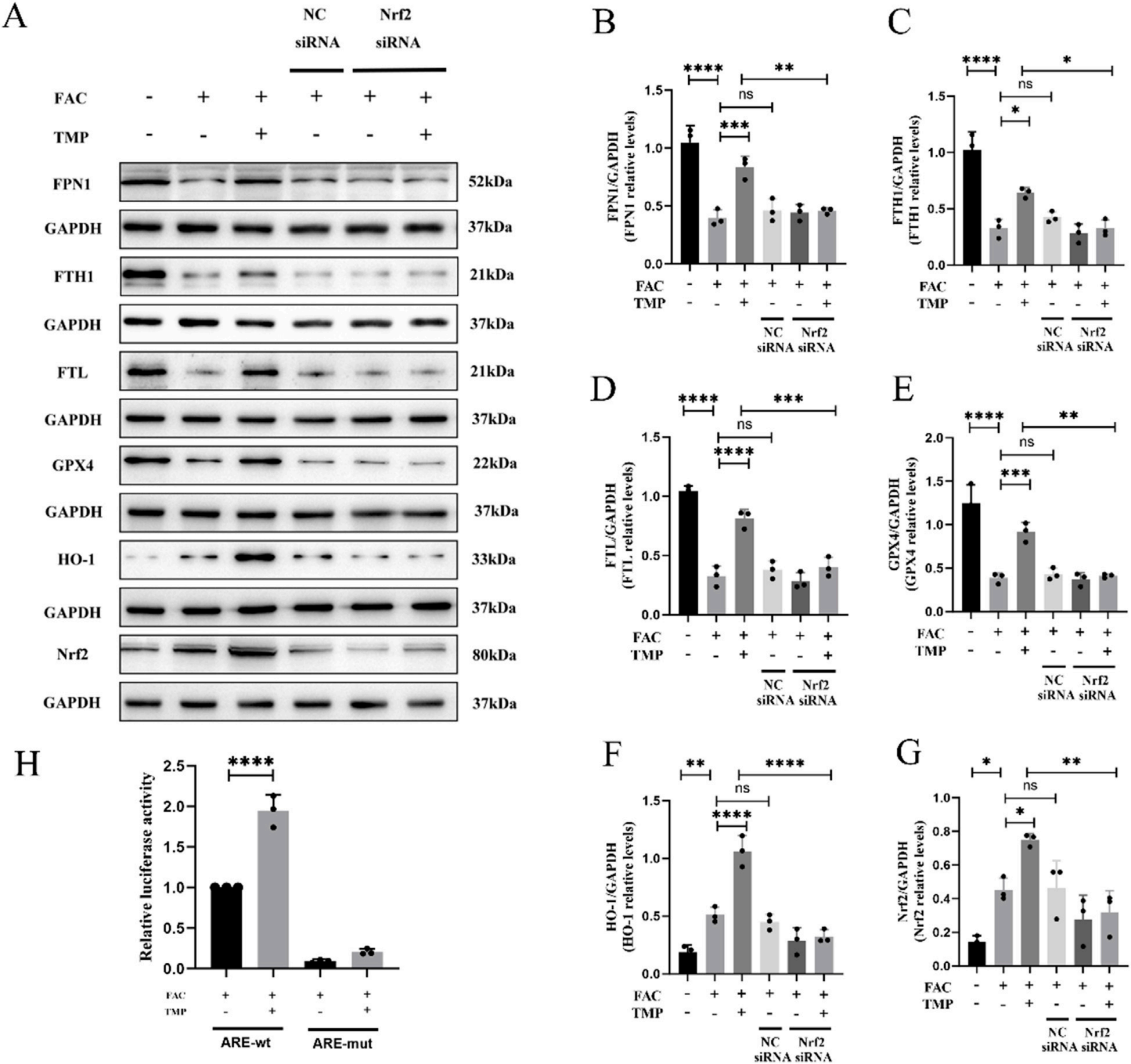
TMP inhibits ferroptosis in VSC4.1 cells by regulating iron metabolism through the NRF2/ARE pathway. **(A)** Western blot and **(B–G)** relative FPN1, FTH1, FTL, GPX4, HO-1, and NRF2 levels in each group (n = 3, statistical analysis was performed with a one-way ANOVA and LSD test, FPN1: F (5, 12) = 25.95, P < 0.0001, FTH1: F (5, 12) = 29.69, P < 0.0001, FTL: F (5, 12) = 54.92, P < 0.0001, GPX4: F (5, 12) = 34.64, P < 0.0001, HO-1: F (5, 12) = 37.65, P < 0.0001, NRF2: F (5, 12) = 11.12, P = 0.0004. *: P < 0.05, **: P < 0.01, ***: P < 0.001, ****: P < 0.0001, ns: the difference was not statistically significant). **(H)** Dual-luciferase reporter gene assay for detection of NRF2 transcriptional activity in each group (n = 3, statistical analysis was performed with a one-way ANOVA and LSD test, F (3, 8) = 221.5, P < 0.0001. ****: P < 0.0001). The data represent means ± SD.

FTH, FTL and FPN1 were the key marker in iron metabolism. GPX4 and HO-1 were a marker of ferroptosis with antioxidant effects. The expression of all these proteins is affected by the transcriptional regulatory effects of Nrf2. We found that TMP promoted the expression of iron metabolism markers FPN1, FTH1, and FTL in ferroptosis cells. TMP also elevated the expression of GPX4 and HO-1, the anti-lipid peroxidation marker. Following the application of NRF2 siRNA to reduce NRF2 expression, the effect of TMP on the expression of iron metabolism molecules was reversed. According to the WB results, it was found that the ferroptosis group had lower expression of iron metabolism markers FPN1, FTH1, and FTL than the control groups. The ferroptosis markers HO-1 and GPX4 were expressed less in the ferroptosis group. Silencing of NRF2 reversed the upregulation of iron metabolism markers and ferroptosis markers by TMP (Figures 6A–G). A dual luciferase reporter gene assay suggests that TMP increased the transcriptional activity of NRF2 and facilitated the binding of NRF2 to the ARE promoter sequence (Figure 6H). It is concluded that TMP promotes the transcriptional activity of the NRF2/ARE pathway and regulates iron metabolism to inhibit ferroptosis.

## 4 Discussion

TMP is a natural alkaloidal ingredient derived from plants. The potential of TMP as a neuroprotective medicine lies in its capacity to cross the blood-brain barrier and enrich the central nervous system with minimal adverse effects or cytotoxicity (Kao et al., 2006; Lv et al., 2012). Researchers have conducted preliminary explorations of TMP’s cytoprotective effects. Zhang et al. (2021) found that TMP inhibits inflammation and protects the blood-spinal cord barrier by regulating microglia’s polarization. According to the research of Duan et al. (2020), TMP is also shown to inhibit lipopolysaccharide-induced apoptosis. However, more research is necessary to understand the biological mechanism of TMP. In our study, neuron apoptosis was reduced in the SCI rats after TMP treatment, and the trend of secondary injury was alleviated. Moreover, TMP treatment improved motor function in rats with SCI. The aforementioned studies also came to similar conclusions. The rats with SCI showed a tendency for spontaneous motor function recovery, and 42 days after injury, their hind limbs could occasionally support their own weight. From the 7th day of treatment with TMP, the rats’ hind limb motor function was substantially better compared to the SCI group. Compared with the SCI group, the TMP treatment increased the amounts of neurons and the integrity of the spinal cord structure. As a result, it is believed that TMP is able to inhibit programmed cell death to promote neurological function recovery.

Ferroptosis is a programmed cell death (PCD) mediated by the accumulation of iron ions and the Fenton reaction. In the last decade, researchers have discovered three central biological mechanisms that regulate ferroptosis (Stockwell, 2022): (1) PUFA and lipid metabolism pathway, (2) ROS-induced peroxidation damage, and (3) Iron metabolism pathway. Increasing evidence suggests that ferroptosis has a close connection with neurological injury, degenerative diseases, and ischemia-reperfusion injury (Zhang et al., 2022). Unlike other cells and tissues, neurons and glial cells possess high levels of PUFA, high oxidative metabolic activity, high ROS metabolites, and low antioxidant capacity (Rosales-Antequera et al., 2022). This suggests that neurons are susceptible to ferroptosis after SCI. In our investigation, the spinal cord in the SCI group had structural damages in the mitochondrial membrane characteristic of ferroptosis, and TMP inhibited mitochondrial damage. This could be due to iron overload causing the Fenton reaction to produce excess ROS, which mediates programmed cell death by disrupting cell membranes (Speer et al., 2013; Sumneang et al., 2020). We also discovered an increase in oxidatively active molecules after SCI, such as ROS and MDA. A higher level of GPX4 and HO-1 expression was observed in the TMP group of rats compared to the SCI group and TMP also inhibits the accumulation of ROS. GPX4 and GSH form an antioxidant system that can reduce lipid peroxides to lipid alcohols. HO-1 may exert antioxidant effects by regulating the balance of heme and nonheme iron (Li et al., 2017). Therefore, we can conclude that TMP inhibits ferroptosis in SCI and exerts neuroprotective effects.

SCI results in an imbalance in the unstable iron pool (Fang et al., 2023). Excess iron produces ROS through the Fenton reaction, eventually leading to ferroptosis (Manz et al., 2016). Hence, we hypothesized that modulating iron metabolism could inhibit ferroptosis in SCI. Iron metabolism is achieved primarily through the regulation of iron storage and export. A ferritin subunit called ferritin heavy chain 1 (FTH1) can bond with ferritin light chain 1 (FTL), converting Fe2+ to Fe3+ and storing it in ferritin, which regulates the intracellular unstable iron pool (Chen et al., 2020b). Studies have shown that mice knocked out of the FTL gene exhibit neuronal and multisystemic imbalances in iron metabolism (Li et al., 2015). Iron accumulation in the nervous system in neurodegenerative diseases is also caused by defects in the human FTL gene (Cozzi et al., 2013). Similarly to ferritin, ferroportin (FPN1) prevents iron overload in cells by controlling intracellular nonheme iron export (Katsarou and Pantopoulos, 2020).

The Nrf2 pathway is a key pathway that regulates oxidative stress response and is closely related to the pathological process of spinal cord injury (Xiao et al., 2024). As a result of endogenous nuclear translocation, Nrf2 regulates the transcriptional activity of endogenous antioxidant enzymes. In addition, Nrf2 binds to ARE to regulate the expression of a large number of downstream factors. These downstream factors are closely related to apoptosis, inflammatory damage and iron metabolism.

*In vivo* experiments, we found that rats with SCI had higher iron contents in their spinal cord tissues. TMP enhanced the levels of NRF2 and proteins associated to iron metabolism. NRF2 expression in the nucleus was increased in TMP group. It was shown that intracellular iron storage and iron export are regulated by the NRF2/ARE pathway (Kerins and Ooi, 2018). Iron metabolism genes are stimulated by the transcription factor NRF2 by binding to the antioxidant response elements in their promoter regions (Chen et al., 2022). From this, we hypothesized that TMP can regulate iron metabolism via the NRF2/ARE pathway.

To validate the regulation mechanism of TMP on iron metabolism, a ferroptosis model that mimics iron overload was constructed *in vitro*. NRF2 siRNAs have been used to manipulate the downregulation of NRF2. We observed that TMP induced the upregulation of antioxidant and iron metabolism markers. The regulatory effect of TMP on iron metabolism was inhibited after the down-regulation of NRF2. Furthermore, consistent with the results observed *in vivo* experiments, TMP could inhibit ferroptosis in the iron overload model, whereas downregulation of NRF2 suppressed the neuroprotective effects of TMP. This suggests that TMP promotes NRF2 binding to the ARE promoter sequence. Thereby promoting the transcription and translation of proteins related to iron metabolism.

Consequently, we conclude that TMP can regulate iron metabolism through the NRF2/ARE pathway and inhibit ferroptosis in neuronal cells.

## 5 Conclusion

**FIGURE 7:**
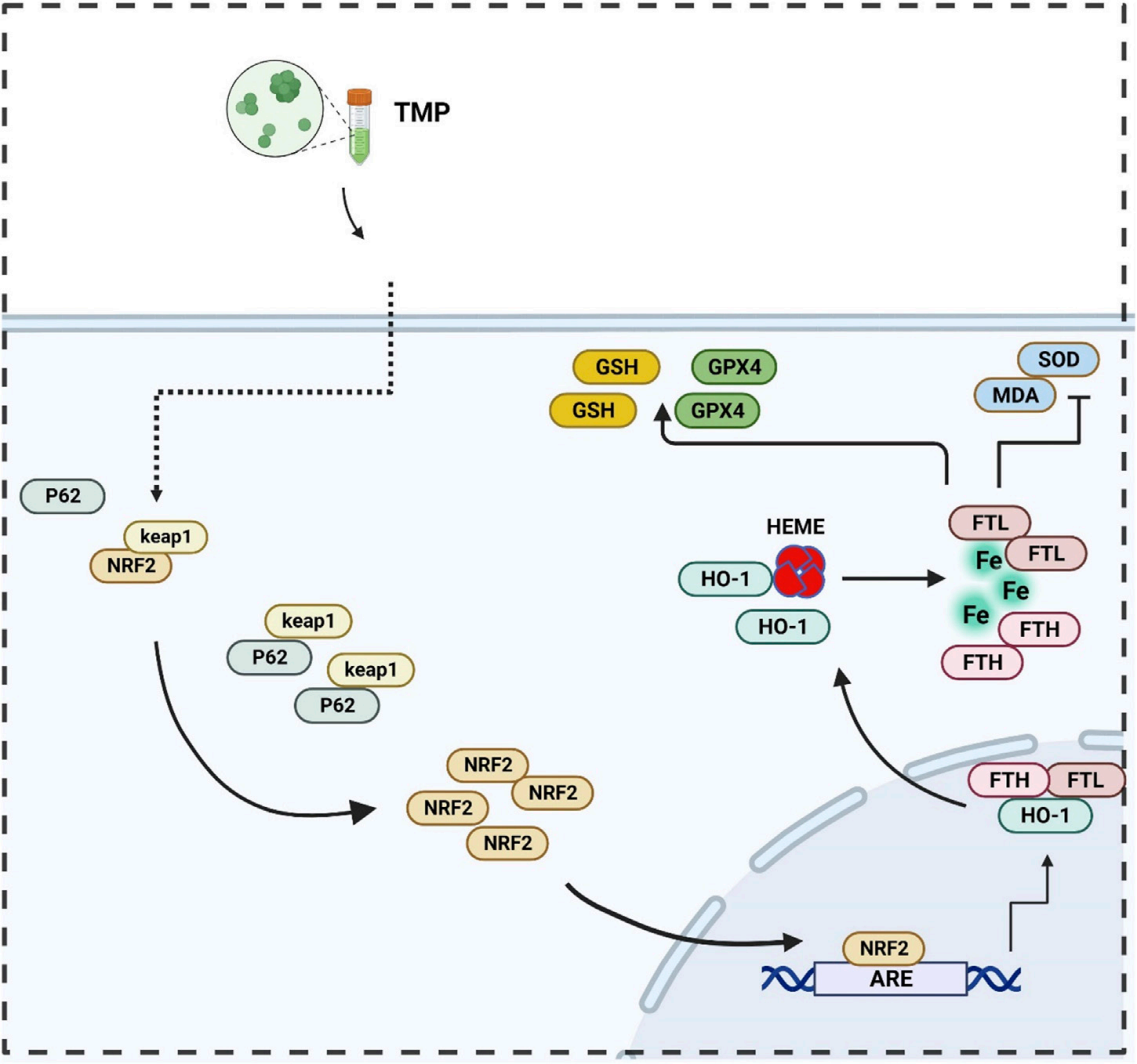
Illustration of TMP inhibits ferroptosis in SCI by regulating iron metabolism through the NRF2/ARE pathway.

**FIGURE 8:**
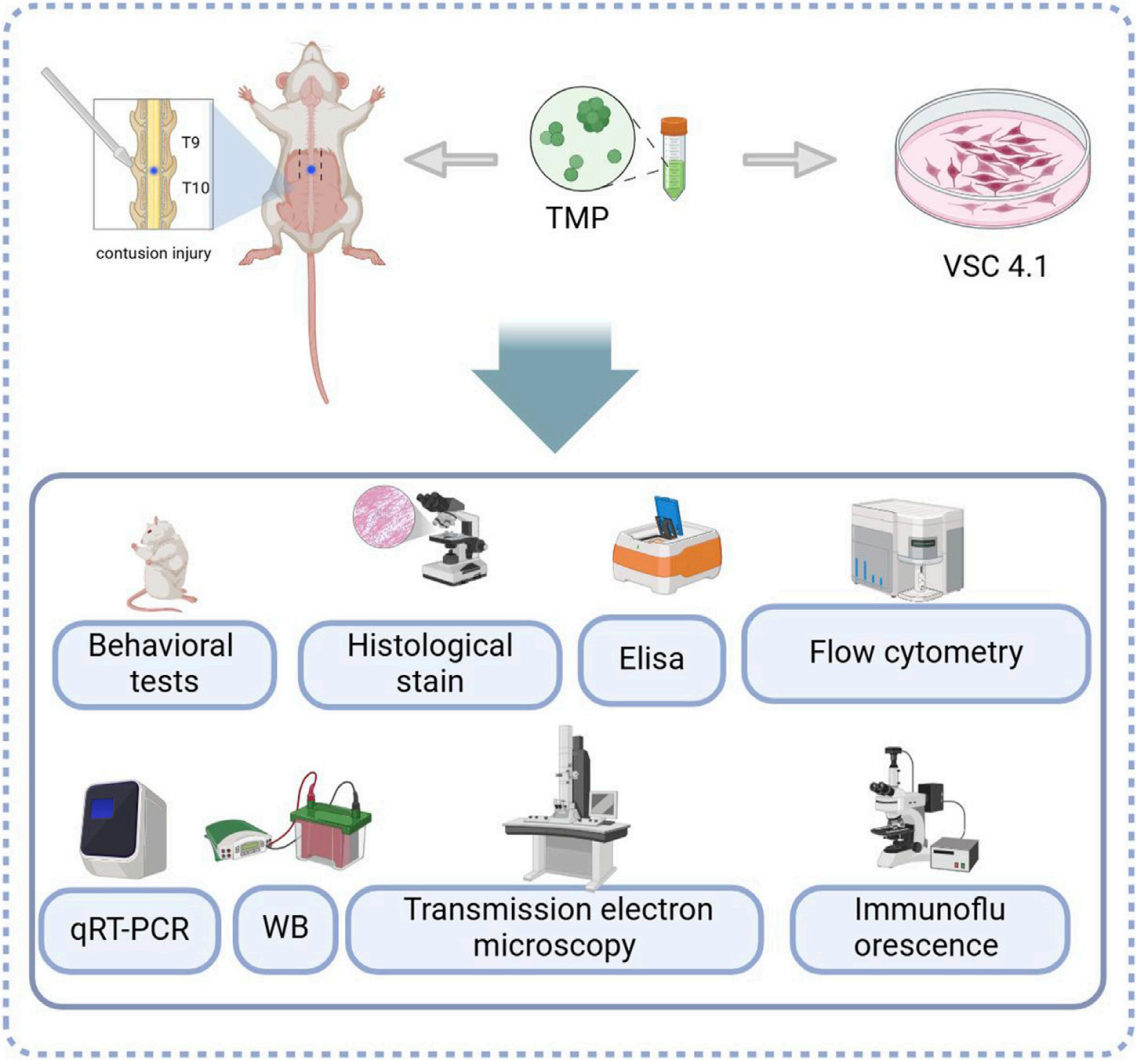
The experimental models and treatments used and analyses performed.

In summary, a regulatory effect of TMP on the NRF2/ARE pathway was found in both *in vitro* and *in vivo* experiments. Promoting transcription and translation of iron metabolizing and antioxidant molecules (Figures 7, 8). Our study explored the inhibitory effect of TMP on ferroptosis from the iron metabolism pathway and provided new ideas for the treatment of SCI.

## Data Availability

The original contributions presented in the study are included in the article/supplementary material, further inquiries can be directed to the corresponding authors.
